# Gender Differences in Developing Biomarker-Based Major Depressive Disorder Diagnostics

**DOI:** 10.3390/ijms21093039

**Published:** 2020-04-25

**Authors:** Mike C. Jentsch, Huibert Burger, Marjolein B. M. Meddens, Lian Beijers, Edwin R. van den Heuvel, Marcus J. M. Meddens, Robert A. Schoevers

**Affiliations:** 1 Brainscan BV, 7418 AH Deventer, The Netherlands; 2 Department of Psychiatry, University Medical Center Groningen, University of Groningen, 9713 GZ Groningen, The Netherlands; 3 Department of General Practice, University Medical Center Groningen, University of Groningen, 9713 GZ Groningen, The Netherlands; 4 Interdisciplinary Center Psychopathology and Emotion Regulation (ICPE), Department of Psychiatry, University Medical Center Groningen, University of Groningen, 9713 GZ Groningen, The Netherlands; 5 Department of Mathematics and Computer Science, Eindhoven University of Technology, 5612 AZ Eindhoven, The Netherlands; 6 Research School of Behavioral and Cognitive Neurosciences, University of Groningen, 9713 AV Groningen, The Netherlands

**Keywords:** major depressive disorder, gender, biomarker panel, ELISA, diagnostic methods, quantile-based prediction, bio depression score

## Abstract

The identification of biomarkers associated with major depressive disorder (MDD) holds great promise to develop an objective laboratory test. However, current biomarkers lack discriminative power due to the complex biological background, and not much is known about the influence of potential modifiers such as gender. We first performed a cross-sectional study on the discriminative power of biomarkers for MDD by investigating gender differences in biomarker levels. Out of 28 biomarkers, 21 biomarkers were significantly different between genders. Second, a novel statistical approach was applied to investigate the effect of gender on MDD disease classification using a panel of biomarkers. Eleven biomarkers were identified in men and eight in women, three of which were active in both genders. Gender stratification caused a (non-significant) increase of Area Under Curve (AUC) for men (AUC = 0.806) and women (AUC = 0.807) compared to non-stratification (AUC = 0.739). In conclusion, we have shown that there are differences in biomarker levels between men and women which may impact accurate disease classification of MDD when gender is not taken into account.

## 1. Introduction

Major depressive disorder (MDD) is a major cause of disability, economic burden and mortality around the world [1]. Appropriate treatment early in the course of the disease has been shown to improve prognosis [2] but requires timely and accurate diagnosis.

Traditionally, the diagnosis of major depressive disorder is based on a clinical interview in which subjectively experienced and in part observable symptoms are identified and categorized according to the Diagnostic and Statistical Manual of Mental Disorders (DSM) or the International Classification of Diseases (ICD) [3,4]. However, the diagnosis of major depressive disorder as produced by these systems is a syndrome diagnosis that harbors high heterogeneity and shows considerable overlap with other psychiatric disorders and physical disease [5]. For example, it is difficult to distinguish MDD from disorders like bipolar disorder [6] and generalized anxiety disorder [7]; an objective laboratory test based on specific biological mechanisms involved in MDD would therefore be very helpful to improve early identification of patients with MDD.

The identification of biomarkers associated with MDD holds great promise for such a test [5,8]. These biomarkers reflect alterations in the neuro-immune and stress system, neuroplasticity, mineral homeostasis, oxidative stress, endothelial function and other mechanisms [5,9,10,11,12,13]. To date, however, no test based on a single biomarker has been developed because individual biomarkers cover only part of these mechanisms and consequently lack sufficient discriminative power [5].

By using multiple markers, the discriminative power of a test is likely to be increased by covering a broader range of the biological processes accompanying MDD [5,14,15]. Nevertheless, discriminative power may still vary depending on individual patient characteristics [5,15,16,17,18].

One such characteristic is gender. MDD is two times more common in women than in men [19], a difference that has been attributed to both psychological and biological differences [20]. Biological explanations may be found in differences in sex hormones, the immune system, serotonergic activity, salivary cortisol levels and structural and functional brain differences [21,22]. All of these may have an impact on the discriminatory power of potential biomarkers for MDD.

To assess whether gender modifies the discriminatory power of potential biomarkers for MDD, we performed a cross-sectional study with four steps. First, we assessed gender differences in serum and urine levels of MDD associated biomarkers, irrespective of disease. Second, we studied overall and gender-specific associations of individual biomarker levels with MDD. Third, we investigated the prediction of MDD disease presence by a panel of biomarkers for MDD. Fourth, we examined whether the prediction was affected by stratification by gender.

## 2. Results

### 2.1. Demographic Characteristics

Demographic and clinical parameters are presented in Table 1. As a result of matching, there was no difference between the mean age of controls and MDD participants.

Mean body mass index (BMI) was slightly higher in the MDD than in the control group, and this difference was statistically significant (Table 1). The average number of symptoms (range) was 7.38 (0–9) for males and 7.73 (0–9) for females. Medication was more prevalent in MDD cases than in controls, but no substantial differences between men and women were observed. Medicines used belonged to one of the following categories; neuropsychotropic, cholesterol, cardiovascular, blockers, immune system, metabolic, corticosteroids, (para)sympathetic and other types of medication. Various participants used multiple forms of medications.

### 2.2. Gender Differences in Biomarker Levels Irrespective of MDD Status

Serum and urine biomarker concentrations for men and women in the total cohort are presented in Table 2. Out of 28 biomarkers analyzed, concentrations of nine biomarkers in serum significantly differed between men and women and twelve in urine. In serum, concentrations of BDNF total, endothelin and TNF receptor 2 were significantly higher in men, whereas concentrations of alpha-1-antitrypsin, apolipoprotein A1, cortisol, leptin, prolactin and resistin were significantly higher in women. In urine, concentrations of alpha-1-antitrypsin and midkine were significantly higher in males, whereas concentrations of aldosterone, calprotectin, cGMP, cortisol, HVEM, isoprostane, leptin, myeloperoxidase, resistin and substance P were significantly higher in females. From all significant biomarkers, the effect sizes were the highest for leptin within serum and cGMP within urine. The lowest effect sizes were found for alpha-1-antitrypsin within urine and BDNF total within serum.

### 2.3. Gender Differences in Biomarker Levels According to MDD Status

From all MDD cases, one can assume that they retain their MDD status irrespective of the day of sample drawing (See Supplementary Figure S1 for the Kaplan–Meier analysis). Mean biomarker concentrations according to MDD status are presented in Table 3 with and without gender stratification. Mann–Whitney U and Levene’s test values are presented in Supplementary Table S1.

In the total population, concentrations of eight biomarkers in serum and five biomarkers in urine were significantly different between MDD and controls. Resistin was the only marker that was significantly different in both serum and urine. When a gender stratification is applied, seven biomarkers in serum and four in urine, in men, differed between MDD and the control group. Among women, four biomarkers were different between MDD and the control group within serum, and another four biomarkers differed in urine. From all biomarkers identified by applying gender stratification, six biomarkers were only identified after gender stratification and were not identified as significant during the initial analysis. There were three biomarkers (apolipoprotein A1, thromboxane B2, and BDNF total) in serum and three biomarkers (calprotectin, cortisol, α1-antitrypsin) in urine. All serum markers were significant for men. From the urine biomarkers, calprotectin was significant within women, whereas cortisol and α1-antitrypsin were only significant within men.

A comparison of biomarker levels between men and women with MDD as presented in Table 4 revealed that out of 28 biomarkers, levels of 16 biomarkers were significantly different, seven within serum and nine in urine.

### 2.4. Gender Stratification and Biomarker Panel Selection with QBP

Table 5 represents quantile-based prediction (QBP) identified relevant biomarkers with and without stratification. Twenty-eight out of 29 biomarkers were deemed relevant in the non-stratified group. When stratified for gender, the QBP method identified different sets of relevant biomarkers for each gender separately. Endothelin and leptin within serum, and myeloperoxidase and midkine within urine, seemed to be more involved in women’s MDD pathophysiology, whereas apolipoprotein A1, EGF and myeloperoxidase within serum and cortisol, substance P and thromboxane B2 seemed to be more involved within men′s MDD pathophysiology. From all biomarkers identified within the stratified groups by QBP, endothelin (serum), myeloperoxidase (urine) and midkine (urine) were only identified by QBP and not previously using the Mann–Whitney test.

### 2.5. BDS and Biomarker Panel Performance in the Total Group and That Stratified for Gender

**Figure 1:**
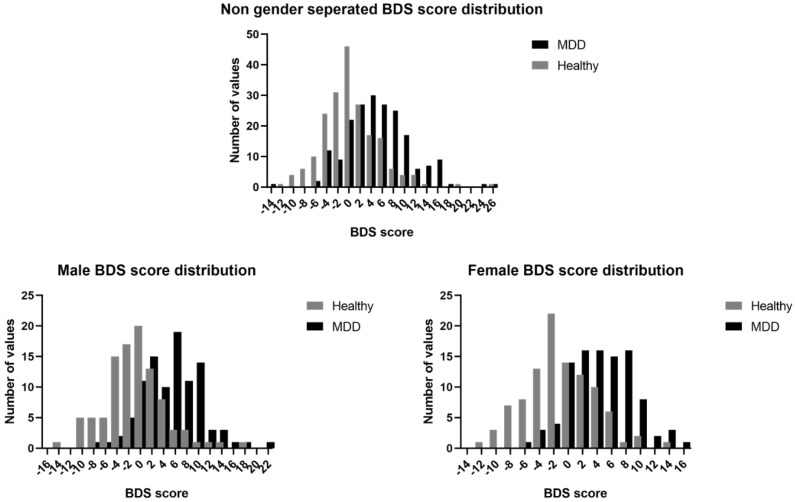
Bio depression score distribution with and without gender stratification. Each generated bio depression score (BDS) is based on all the actively contributing biomarkers within the quantile-based prediction (QBP) for each configuration.

The Bio Depression score BDS was calculated based on the optimized inclusion/exclusion criteria for relevant biomarker tails. For the total group, criteria were set at 20%/6%, criteria for men were set at 17%/6% and for women at 17%/7%. Permutation analysis with all 29 biomarkers (including BMI) showed significant BDS discrimination for the non-stratified (*p* < 0.0001) as well as the stratified biomarker panels (men *p* < 0.01, women *p* < 0.05). Figure 1 depicts the distribution of the bio depression score (BDS) in the total study group as well as that stratified for gender. There was no significant difference between the mean BDS scores of all MDD groups (non-stratified and stratified). Regression analysis showed no direct interaction between gender, BDS score and MDD status. However, gender seems to have a small confounding effect as its inclusion into the regression model increased the odds ratio of the BDS score from 1177 (CI: 1.125–1.232) to 1205 (CI: 1.147–1.267).

### 2.6. Disease Classification with ROC Analysis

**Figure 2:**
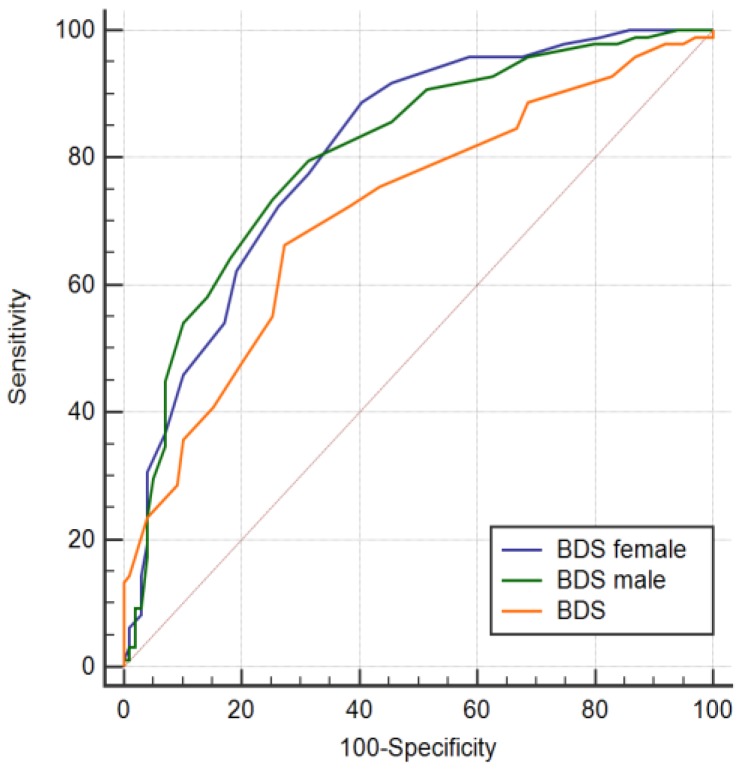
Receiver operator characteristic (ROC) curves of the bio depression score obtained from the combined active serum and urine biomarkers determined with the quantile-based prediction with and without gender stratification and after randomization. AUC_BDS_ =0.739 (sensitivity= 74%, specificity= 66%) with 22 contributing biomarkers, AUC_Male_ = 0.807 (sensitivity = 80%, specificity = 69%) with 26 contributing biomarkers and AUC_Female_ = 0.806 (sensitivity = 89%, specificity = 59%) with 22 contributing biomarkers.

Without gender stratification, an Area Under Curve (AUC) of 0.739 was calculated. Subsequent gender-specific AUC calculation (with the BDS based on the total group) resulted in an AUC of 0.760 for men and 0.751 for women. Stratified for gender and based on gender-specific criteria in the BDS, the AUC in men (AUC = 0.806) and women (AUC = 0.807) increased, although not significantly (total group BDS vs. men BDS, *p* = 0.089; total group BDS vs. women BDS, *p* = 0.090). Receiver operator characteristic (ROC) curves of the non-stratified BDS and gender stratified groups are visualized in Figure 2.

Without gender stratification, out of participants classified by BDS as having MDD, 37% were correct whereas participants classified by BDS as control, 33% were correct. Correct identification increased when gender stratification was applied. BDS correctly classified MDD within 40% of men and 44% of women. The number of false positives increased within the women group, whereas that within the men group decreased compared to no gender stratification. The percentage of false negatives decreased when gender stratification was applied.

## 3. Discussion

The aim of the current paper was to investigate the effect of gender on the discriminative power of potential biomarkers for MDD in serum and urine. First, we focused on gender differences in serum and urine levels of MDD-associated biomarkers, irrespective of MDD, and found that there are several differences in biomarker levels between men and women. Second, we focused on associations of biomarker levels within MDD with and without gender stratification. Without gender stratification, eight biomarkers in serum and five biomarkers in urine were significantly different between the MDD and control group. When gender stratification was applied, differences concerned seven biomarkers in serum and five in urine in men, whereas in women, differences regarded four in serum and four in urine. Six of those biomarkers were only identified as different between MDD and control after applying a gender stratification. Last, we investigated the effects of gender stratification on MDD disease classification by a panel of biomarkers using a novel statistical method called quantile-based prediction. This analysis revealed that when the QBP was applied for each gender separately, the predictive accuracy improved as evident from an increase in AUC from 0.739 calculated for the total population to 0.805 in men and 0.807 in women, although this increase was not statistically significant. To our knowledge, this is the first study to investigate the modifying effects of gender on the discriminative power of serum- and urine-based biomarkers for MDD.

Before we can accept the findings of our study, some limitations need to be addressed. One of the major issues in the development of biomarker panels is that the performance of specific biomarkers panels is measured against the disease state, which in turn is determined by the use of diagnostic instruments. Although all diagnostic instruments are in principle clinically valid, they have their limitations with respect to correctly diagnosing psychiatric disorders like MDD, which makes a biomarker performance assessment as good as the reference tool which is used for the diagnosis. Within the current study, we used the Mini-International Neuropsychiatric Interview (MINI), which is a valid instrument for diagnosing DSM-IV disorders [23]. The sensitivity of the MINI is highest for MDD, albeit with a high false-positive scoring rate [23,24,25], which increases the chance of falsely linking biomarkers to MDD. To reduce the possibility of false-positive MDD classification by the MINI, we selected participants with moderate-to-severe MDD as defined by the presence of at least some disability (i.e., staying in bed due to psychopathology, being unable to do normal activity/work at all, being unable to do the normal amount of activity/work or being unable to have normal quality in activity/work. Another important factor is the time between the administration of the questionnaire and drawing biological samples. For a direct correlation, a limited time between the questionnaire and sample drawing is essential because levels of biomarkers are to be related to the disease state at that moment. However, within the current study, the time between the MINI and drawing of biological samples varied greatly, including in one participant, for which this time lag exceeded 512 days. The difference in time makes it more difficult to directly correlate biological markers to disease status, especially for subjects with a larger time difference. In these patients, one can question disease status based on the duration of MDD [26]. However, in combination with data on the prevalence of MDD [27], we estimated the chance of false positives in the cohort over time by using a Kaplan–Meier survival model. Results showed that from all participants initially diagnosed with MDD, 90% are still depressed at the time of biological sample drawing. Although 90% seems sufficiently high to assess correlations between biomarker data and disease state, the 10% of misdiagnoses may have had an influence on the performance of our biomarker panel. It is, however, unlikely to have had an effect on the 28 biomarkers which we identified to be significantly different between men and women.

With respect to the observed biomarker differences between men and women irrespectively from disease status, there are some points worth considering. For this analysis, we used biomarker data from the combined group of controls and MDD patients, which could have skewed the individual differences in biomarker levels leading to detecting gender differences, but which were actually absent. Other factors that may have influenced the differences between men and women and also between control and MDD are BMI, age and smoking. For both factors, it is known that they can influence biomarker levels: leptin levels, for example, increase with increasing BMI [28], whereas age affects multiple biomarkers of the Hypothalamic Pituitary Adrenal axis (HPA axis), the immune system and neurotrophic factors, leading either to an increase or decrease of levels [29,30,31]. Although not explicitly investigated within the current study, the previously observed positive correlation between BMI and leptin levels seems to be supported by our data showing that higher BMI and higher leptin levels within serum are associated with MDD (data not shown). The effects of age on biomarker levels were not investigated within the current scope of the study, but participants were matched for age, therefore limiting its potential influence. At the same time, this procedure may limit extrapolation to a larger age group. As with age, smoking is another factor which was currently not within the scope of the study but could potentially explain differences between men and woman as well as between controls and MDD. It was previously shown that tobacco use increases plasma levels of BDNF within MDD participants as compared to nonsmoking MDD participants [32]. Next to age, BMI and smoking, the use of prescription medicine may have altered differences in biomarker levels. A recent study has shown that prescription drugs can affect 1 to 250 different proteins/biomarkers and that even after correction for other covariates such as gender, age and smoking, medicine use still accounts for a substantial part of the observed variance in biomarker levels at the population level [33]. Another factor which could have had substantial effect on the performance of our biomarker panel is the Caucasian descent of our study population, which may limit extrapolation to other ethnic groups. In a recent study, van Buel et al. (2019) showed that the AUC varies using the same set of biomarkers when stratified for Caucasians and other ethnicities [15].

Previous research showed that one of the major issues in developing suitable biomarker panels is finding biomarkers that have, when combined, sufficient discriminative power to be of diagnostic value [5,34]. Ideally, these are biomarkers which are associated with a specific biological pathway. However, due to the heterogeneous nature of MDD, it is hard to determine specific biological markers for MDD, leading to discussions about the validity of a potential biomarker (and by extension biomarker panels). With respect to the currently investigated biomarker panel, biomarkers were included which were carefully selected and covered various biological mechanisms associated with MDD (see Supplementary Table S2). Still, only a subset of biomarkers could be associated with MDD within the study population. It could be that within the current population only a subset of biological pathways were actively involved. The association between individual biomarkers and MDD is, however, not solely dependent on specific biological pathways but also on gender. By applying gender stratification, a different pattern of associated biomarkers emerged which, interestingly, was associated with different biological pathways. Our data suggest that biological mechanisms such as the HPA axis/stress, neuroplasticity, endothelial dysfunction and neuro immune-inflammation are more likely to be involved in male MDD pathophysiology, whereas a strong immune response and endothelial dysfunction/oxidative stress are more likely to be involved in female MDD pathophysiology. Gender-specific pathophysiology patterns are also supported by recent studies showing gender-specific neuroimmune dysregulation [35], male-specific HPA axis dysregulation within MDD related to alcohol abuse [36], and gender-specific differences at the level of immune responses [37].

After gender stratification, the AUC increased in our study. Albeit not significantly, the increase of AUC after stratification suggests a better biomarker performance, which is also evidenced by the change in contributing biomarkers. Within both genders, less biomarkers contributed more to a higher AUC, suggesting that these biomarkers more closely reflect the underlying biological pathways. Furthermore, a recent analysis performed by our group involving a larger cohort showed that after gender stratification, the AUC increases with less contributing biomarkers compared to the non-gender-stratified group. The difference in AUC remained after performing a 5-fold cross-validation (see Supplementary Table S3) [38]. An advantage of the new advanced statistical method we used is that we were able to utilize the full informative potential of our biomarkers with respect to underlying biomarker dynamics and the contribution to disease status, thereby limiting the effects of the high variability in biological pathways involved in MDD. The application of advanced statistical methods is not new: others have shown that by utilizing potential information buried within biomarker data, one can develop biomarker panels with high discriminative power [5,15]. This concept of identifying contributing biomarkers is in line with our QBP method, which showed not only that with gender stratification, different patterns of contributing biomarkers can be identified, but that this method also eliminated non-relevant biomarkers which were previously identified as biomarkers associated with MDD and vice versa.

In future studies utilizing QBP, transforming biomarker data before applying QBP might positively affect performance. Papakostas et al. (2013) have previously shown that data transformation improved their algorithm, resulting in the detection of MDD with high sensitivity and specificity [17]. The QBP method we used can be further optimized by selecting only relevant tails (disease or non-disease-associated) from one biomarker, thereby reducing potential background noise which could interfere with the analysis. Still, without performing extensive data transformations, our method could quite well distinguish between MDD and controls.

In summary, we have shown that gender differences play an important role in not only biomarker identification but also the development of biomarker panels for MDD. Gender stratification increased the discriminative power of our QBP biomarker panel. Further, several points were mentioned which could further increase the performance of our biomarker panel.

## 4. Materials and Methods

### 4.1. Study Design

The present study was a cross-sectional analysis of participant data and biological specimens (serum and urine) from the Lifelines Cohort Study and biobank [39,40]. Lifelines is a large multi-disciplinary prospective population-based cohort study examining risk factors for multifactorial diseases among 167,729 persons living in the north of the Netherlands. It employs a broad range of investigative procedures in assessing the biomedical, socio-demographic, behavioral, physical and psychological factors which contribute to the health and disease of the general population, with a special focus on multi-morbidity and complex genetics. Data and biological specimens are collected every five years. Baseline assessments took place between 2006 and 2013. The present analysis was performed using data from the first two waves. The study complied with the principles enunciated in the Declaration of Helsinki and was approved by the medical ethics review committee of the University Medical Center Groningen. Informed consent was acquired from all participants.

### 4.2. Study Population

The study population consisted of 200 participants with a current MDD diagnosis and 200 1:1 age- and gender-matched controls from the total Lifelines cohort. Current MDD was assessed according to DSM-IV-TR criteria with a standardized diagnostic interview based on the Mini-International Neuropsychiatric Interview (MINI). Missing data were allowed when it did not interfere with the establishment of a diagnosis (e.g., no data were required for the additional symptoms of MDD when the core criteria were both absent). We selected only MDD subjects with at least one day of disability a month, according to the four disability questions from the MINI (i.e., staying in bed due to psychopathology, being unable to do normal activity/work at all, being unable to do the normal amount of activity/work or being unable to have normal quality in activity/work). Medication use was measured by asking the participants to bring their medication to the baseline interview where the research assistant noted the corresponding Anatomic Therapeutic Chemical code (ATC). Controls were selected when they reported no MDD symptoms and did not qualify for another MINI diagnosis. Exclusion criteria included non-Caucasian descent, pregnancy, self-reported substance abuse disorder (question: did you have contact with addiction care in the past 12 months) or self-reported physical illness (i.e., kidney problems, cardiovascular problems, cancer, diabetes type 1 or 2, and thyroid problems).

### 4.3. Biomarker Selection and Measurements

The selection of biomarkers was based on a previous study by our group [15] which identified seven biomarkers in serum and eleven in urine. These biomarkers were supplemented with an additional seven biomarkers in serum and three in urine, based on a literature search using various combinations of the terms MDD, depression, biomarkers, urine, serum and pathophysiology. Table 6 shows an overview of biomarkers selected for the present study. Supplementary Table S3 shows an overview of the biological mechanisms in which they are involved. The concentration of the biomarkers as measured in urine are all expressed as the amount of biomarker relative to the amount of creatinine, calculated as the ratio between the concentration in urine of the biomarker divided by the concentration of creatinine. In addition, BMI was added as an extra non-matrix based biomarker due to the high association between BMI and MDD [41].

Biomarker levels in serum and urine were determined by Enzyme-Linked Immunosorbent Assays (ELISA). ELISA kits were obtained from various vendors (see Table 6). ELISA procedures were performed using specific Standard Operating Procedures (SOPs) in which all experimental variables are recorded to generate full experimental traceability for each run. Each SOP was set up according to the manufacturer’s instructions with minor modifications (like adding extra calibrators, altered sample dilutions). An ELISA plate washer (Biorad PW40) was used for all washing steps. TMB absorption measurements were performed on a Microtiter plate reader (Thermo Multiskan Spectrum) at 450 nm using 620 nm as a reference wavelength. Unknown biomarker concentrations were determined by the use of a 4-parameter logistic regression (4-PL) model without weight factors [42]. In short, unknown sample concentrations are calculated based on the optimal fit of the standard curve (based on the Optical Density values) calculated within the 4-PL model. The application of a weight factor can be applied to better fit unknown sample concentrations with a relatively low sample concentration since these samples tend to fit worse compared to samples falling within the middle and upper parts of the curve [42].

Since the ELISAs used are provided at the research and development level, performance parameters such as calibration and reproducibility are not well controlled, potentially leading to biomarker variations when measured over various runs. To account for this, biomarker testing was designed such as to maximally reduce uncontrolled variability by means of measuring all samples in one run for each biomarker. The sample plate positions were randomized for each run.

### 4.4. Statistical Analysis

Descriptive statistics were calculated for demographic and clinical characteristics of the study population according to MDD status and gender. These characteristics were first compared between MDD cases and controls and subsequently within MDD status between men and women.

Mean levels of biomarkers were first compared in the total population between men and women. Thereafter, we assessed differences in these levels according to MDD status in the total group and while stratifying for gender.

Categorical data were compared using chi-square tests, and continuous data including differences in biomarker levels were compared using the non-parametric Mann–Whitney test because they showed a non-normal distribution. The Levene’s test to assess heterogeneity was applied to determine variance differences in each individual biomarker. A Kaplan–Meier analysis was performed in order to investigate the effect of time between sample drawing and the initial diagnosis which in some occasions was more than 30 days.

For the binary classification of MDD presence, a newly developed method called quantile-based prediction (QBP) was used [15]. In short, QBP assigns scores to certain percentiles in the left and right tails of empirical biomarker distributions. Tails in which a shift case versus control is observed (differences between case and control) are assigned a value ≠0, and tails where no difference is found are assigned 0. The further the percentile is removed from the 50th, the higher the absolute value of the score. For relevant tails (tail either represents disease or control) three percentiles (P10, P5 and P1, or P90, P95 and P99) are selected and receive a value of 1, 2 or 3, respectively. For each singular biomarker, a positive or negative score is assigned such that a positive score is associated with MDD and a negative score with control status. The sum of scores for all biomarkers is the Bio Depression Score (BDS) for each participant based on the relevant tails. Next, we performed a receiver operator characteristic (ROC) analysis by relating the score to the presence of MDD for various cut-offs. The AUC was calculated as a cut-off-independent measure of discriminatory power of the score. The next step involved optimization of the selection criteria of relevant biomarker tails by empirically making the criteria for inclusion of relevant biomarkers more or less stringent. This was accomplished by varying the threshold on the exceed ratio (ratio between the dominant and non-dominant group for each of the three percentiles). By varying the threshold at the 90 and 95 percentile and the 5 and 10 percentile, the AUC increased or decreased. The optimal criteria were set at the level where the AUC started to decrease and are expressed as percentages. The AUCs of the total as well as the gender-specific groups were determined based on the BDS score calculated by the QBP method including the total population. For the gender-stratified analysis, the AUCs were determined based on the BDS estimated in men and women separately.

The statistical significance of the discriminative power of the optimal BDS and calculated AUC was assessed by performing a permutation analysis (approx. 2000 permutations) in which the case control/control indicator was randomly distributed over the original biomarkers data generating randomly generated AUCs. Possible interaction between gender and the BDS score in relation to MDD status was assessed by testing the statistical significance of a product term gender*BDS as an independent variable in a binary logistic regression model including the total population. For the assessments of discriminative power, the Youden index was used.

The statistical significance level was set at 0.05, two-sided.

QBP analysis, calculation of ROC curves, the AUC permutation analysis and the accompanying sensitivity and specificity was done in labview (see [15]) and in Medcalc version 18.11.3. Group comparisons were performed within GraphPad prism 8 and IBM SPSS statistics 26. The binary logistic regression was performed with IBM SPSS statistics 26.

## 5. Conclusions

We have demonstrated that at the gender level, numerous biomarker differences can be found not only irrespective of disease but also between controls and MDD patients. We also have shown that some of these biomarker differences are specific to either males or females and that without gender differences taken into account, possible MDD candidate biomarkers can be missed. Next, we have shown that gender differences likely play an important role in biomarker panel development in terms of performance. Selecting for gender increased the performance of our biomarker panel in terms of discriminative power, although not significantly. Our results indicate that there might be a need to focus on gender differences, but more studies are needed to confirm.

## Figures and Tables

**Table 1:** Demographic and clinical characteristics of the study population.

		Control		MDD		*p*-Value
		Men	Women	Men	Women	
Group distribution		100	100	100	100	-
Age (Years)	Mean	42.92	41.68	42.93	41.21	0.926 ^a^
	SD	10.65	10.92	10.69	11.69	
Medication use	Yes	24	31	61	64	<0.0001 ^b^
	No	76	69	39	36	
BMI (kg/m^2^)	Mean	26.05	24.86	27.04	26.89	0.004 ^a^
	SD	3.39	3.93	4.57	5.33	

^a^: Mann–Whitney U test (calculated mean control vs. mean major depressive disorder MDD); ^b^: Chi-square test (2 × 2).

**Table 2:** Gender-specific serum and urine biomarker concentrations in the total study population.

	Biomarkers	Units of Measurment	Males	Females	*p* (MW)	*p* (Levene′s)	Effect Size * r
Serum							
	α1 anti-trypsin	mg/L	547 ± 272	563 ± 249	<0.0001	0.132	0.28
	Apolipoprotein A1	μg/mL	2170 ± 310	2280 ± 325	<0.0001	0.036	0.71
	BDNF free	ng/mL	18.9 ± 6.12	18.3 ± 5.76	0.623	0.567	-
	BDNF Total	ng/mL	36.7 ± 8.54	35.8 ± 8.16	0.574	0.025	0.02
	Calprotectin	μg/mL	2.28 ± 1.48	2.14 ± 1.52	0.167	0.606	-
	Cortisol	μg/dL	17.60 ± 5.87	18.40 ± 6.29	0.045	0.0167	0.26
	EGF	pg/mL	526 ± 171	513 ± 167	0.283	0.829	-
	Endothelin	pg/mL	1.60 ± 0.50	1.51 ± 0.45	0.010	0.336	0.26
	Leptin	ng/mL	67.3 ± 62.1	87.6 ± 60.8	<0.0001	<0.0001	1.13
	Myeloperoxidase	ng/mL	333 ± 198	314 ± 177	0.470	0.477	-
	Prolactin	μlU/mL	16.4 ± 14.9	20.1 ± 18.2	<0.0001	0.005	0.35
	Resistin	ng/mL	13.9 ± 4.45	14.5 ± 5.30	<0.0001	0.013	0.48
	Thromboxane B2	ng/mL	22.0 ± 21.1	21.4 ± 21.1	0.524	0.930	-
	TNF receptor 2	ng/mL	2.26 ± 0.53	2.16 ± 0.50	0.009	0.186	0.21
Urine							
	Aldosterone	pg/mg	476 ± 218	677 ± 329	<0.0001	<0.0001	0.72
	α1 anti-trypsin	ug/mg	32.6 ± 87.6	30.6 ± 70.0	0.013	0.661	0.01
	Calprotectin	ng/mg	23.0 ± 68.3	106 ± 134	<0.0001	<0.0001	0.78
	cGMP	pmol/mg	34.0 ± 12.5	51.1 ± 18.1	<0.0001	<0.0001	1.09
	Cortisol	μg/mg	5.84 ± 3.25	7.11 ± 3.47	<0.0001	0.083	0.37
	HVEM	pg/mg	928 ± 178	10400 ± 214	<0.0001	0.045	0.57
	Isoprostane	ng/mg	0.16 ± 0.05	0.17 ± 0.07	0.291	0.018	0.19
	Leptin	pg/mg	0.37 ± 0.68	2.94 ± 6.63	<0.0001	<0.0001	0.54
	LTB4	pg/mg	40.7 ± 14.6	44.9 ± 22.7	0.057	0.102	-
	Midkine	pg/mg	16.3 ± 23.2	9.39 ± 22.2	<0.0001	0.026	0.30
	Myeloperoxidase	ng/mg	2.30 ± 13.1	7.83± 17.3	<0.0001	0.0008	0.36
	Resistin	ng/mg	0.48 ± 0.36	0.71 ± 0.62	<0.0001	0.001	0.45
	Substance P	pg/mg	43.6 ± 15.1	47.3 ± 15.5	0.004	0.212	0.23
	Thromboxane B2	ng/mg	2.86 ± 2.19	2.90 ± 2.20	0.806	0.573	-

* Effect size was only calculed for significant biomarkers; Urine biomarker concentrations are presented as the creatine corrected concentrations.

**Table 3:** Serum and urine biomarker concentrations with and without gender stratification.

	Biomarkers	Units of Measurement	No Gender Selection			Men			Women		
			MDD	Control	*p*-Value *	MDD	Control	*p*-Value *	MDD	Control	*p*-Value *
**Serum**											
	α1 anti-trypsin	mg/L	547 ± 272	510 ± 195	*p* > 0.05	523 ± 298	471 ± 97.1	*p* > 0.05	575 ± 245	550 ± 252	*p* > 0.05
	Apolipoprotein A1	mg/mL	2170± 310	2170 ± 350	*p* > 0.05	2073 ± 239	2050 ± 340	*p* < 0.05	2270 ± 340	2300 ± 310	*p* > 0.05
	BDNF free	ng/mL	18.9 ± 6.12	17.7 ± 4.82	*p* > 0.05	18.8 ± 6.08	17.8 ± 4.43	*p* > 0.05	19.1 ± 6.21	17.6 ± 5.17	*p* > 0.05
	BDNF Total	ng/mL	36.7 ± 8.54	35.2 ± 7.60	*p* > 0.05	36.8 ± 8.73	35.3 ± 6.35	*p* < 0.05	36.7 ± 8.47	35.0 ± 8.66	*p* > 0.05
	Calprotectin	μg/mL	2.28 ± 1.48	1.82 ± 1.09	*p* < 0.05	2.12 ± 1.15	1.79 ± 0.98	*p* < 0.05	2.42 ± 1.74	1.85 ± 1.20	*p* < 0.001
	Cortisol	μg/dL	17.6 ± 5.87	17.6 ± 5.40	*p* < 0.05	16.3 ± 5.04	17.4 ± 4.44	*p* > 0.05	19.0 ± 6.30	17.8 ± 6.21	*p* > 0.05
	EGF	pg/mL	526 ± 171	479 ± 157	*p* < 0.05	525 ± 179	459 ± 140	*p* < 0.05	527 ± 163	499 ± 170	*p* > 0.05
	Endothelin	pg/mL	1.60 ± 0.50	1.55 ± 0.47	*p* > 0.05	1.63 ± 0.55	1.62 ± 0.46	*p* > 0.05	1.54 ± 0.44	1.47 ± 0.46	*p* > 0.05
	Leptin	ng/mL	67.3 ± 62.1	53.2 ± 49.1	*p* < 0.01	33.9 ± 33.4	30.4 ± 32.7	*p* > 0.05	99.4 ± 66.2	75.8 ± 52.3	*p* < 0.05
	Myeloperoxidase	ng/mL	333 ± 198	282 ± 159	*p* < 0.05	322 ± 202	284 ± 170	*p* > 0.05	347 ± 196	280 ± 148	*p* < 0.05
	Prolactin	μlU/mL	16.4 ± 14.9	18.1 ± 16.9	*p* < 0.05	12.38 ± 7.37	16.4 ± 16.0	*p* < 0.01	20.4 ± 18.9	19.8 ± 17.5	*p* > 0.05
	Resistin	ng/mL	13.9 ± 4.45	12.8 ± 5.04	*p* < 0.01	12.97 ± 4.13	11.5 ± 3.53	*p* < 0.05	14.9 ± 4.57	14.1 ± 5.91	*p* < 0.05
	Thromboxane B2	ng/mL	22.0 ± 21.1	21.2 ± 20.3	*p* > 0.05	25.3 ± 21.9	18.4 ± 15.8	*p* < 0.05	18.8 ± 20.0	23.9 ± 23.7	*p* > 0.05
	TNF receptor 2	ng/mL	2.26 ± 0.53	2.16 ± 0.49	*p* < 0.05	2.29 ± 0.52	2.23 ± 0.51	*p* > 0.05	2.23 ± 0.53	2.09 ± 0.45	*p* > 0.05
Urine											
	Aldosterone	pg/mg	590 ± 300	564 ± 292	*p* > 0.05	504 ± 222	449 ± 211	*p* > 0.05	675 ± 341	679 ± 315	*p* > 0.05
	α1 anti-trypsin	μg/mg	37.0 ± 93.4	37.0 ± 61.5	*p* > 0.05	19.2 ± 55.3	45.8 ± 109	*p* < 0.05	33.1 ± 66.4	28.2 ± 73.4	*p* > 0.05
	Calprotectin	ng/mg	76.0 ± 129	53.4 ± 96.3	*p* > 0.05	26.5 ± 77.0	19.6 ± 58.1	*p* > 0.05	126 ± 149	87.2 ± 114	*p* < 0.05
	cGMP	pmol/mg	44.5 ± 18.3	40.7 ± 17.0	*p* < 0.05	36.1 ± 13.6	32.0 ± 11.0	*p* < 0.05	52.9 ± 18.6	49.3 ± 17.5	*p* > 0.05
	Cortisol	μg/mg	6.71 ± 3.53	6.25 ± 3.29	*p* > 0.05	6.41 ± 3.52	5.27 ± 2.85	*p* < 0.01	7.01 ± 3.51	7.22 ± 3.41	*p* > 0.05
	HVEM	ng/mg	10.4 ± 2.17	9.32 ± 1.76	*p* < 0.01	9.76 ± 1.76	8.80 ± 1.67	*p* < 0.001	11.0 ± 2.36	9.38 ± 1.70	*p* < 0.01
	Isoprostane	ng/mg	0.18 ± 0.06	0.16 ± 0.06	*p* < 0.01	0.17 ± 0.05	0.16 ± 0.05	*p* > 0.05	0.19 ± 0.07	0.16 ± 0.06	*p* < 0.01
	Leptin	pg/mg	1.98 ± 5.74	1.32 ± 3.83	*p* > 0.05	0.41 ± 0.62	0.32 ± 0.72	*p* > 0.05	3.56 ± 7.78	2.32 ± 5.17	*p* > 0.05
	LTB4	pg/mg	45.1 ± 23.6	40.5 ± 13.0	*p* > 0.05	42.2 ± 16.5	39.2 ± 12.3	*p* > 0.05	47.9 ± 28.7	41.8 ± 13.6	*p* > 0.05
	Midkine	pg/mg	12.7 ± 20.5	13.0 ± 25.1	*p* > 0.05	16.7 ± 24.6	15.8 ± 21.7	*p* > 0.05	8.58 ± 14.3	10.2 ± 27.8	*p* > 0.05
	Myeloperoxidase	ng/mg	6.37 ± 20.4	3.76 ± 8.37	*p* > 0.05	3.12 ± 17.7	1.48 ± 5.69	*p* > 0.05	9.62 ± 22.3	6.05 ± 9.87	*p* > 0.05
	Resistin	ng/mg	0.67 ± 0.64	0.51 ± 0.35	*p* < 0.05	0.53 ± 0.43	0.43 ± 0.26	*p* > 0.05	0.82 ± 0.77	0.60 ± 0.40	*p* < 0.01
	Substance *p*	pg/mg	46.6 ± 16.2	44.3 ± 14.5	*p* > 0.05	45.4 ± 16.4	41.8 ± 13.6	*p* > 0.05	47.8 ± 15.9	46.7 ± 15.0	*p* > 0.05
	Thromboxane B2	ng/mg	3.10 ± 2.14	2.67 ± 2.22	*p* < 0.01	3.11 ± 2.12	2.62 ± 2.22	*p* > 0.05	3.09 ± 2.15	2.72 ± 2.22	*p* > 0.05

* For exact statistics see Supplementary Table S1; Urine biomarker concentrations are presented as the creatine corrected concentrations.

**Table 4:** Differences in serum and urine biomarker concentrations between men and women with MDD.

	*p*-Value	
	Serum	Urine
**Aldosterone**	-	0.000
**α1 anti-trypsin**	0.001	0.006
**Apolipoprotein A1**	0.000	-
BDNF free	0.759	-
BDNF total	0.738	-
**Calprotectin**	0.135	0.000
**cGMP**	-	0.000
**Cortisol**	0.008	0.175
EGF	0.831	-
Endothelin	0.261	-
**HVEM**	-	0.000
Isoprostane	-	0.099
**Leptin**	0.000	0.000
LTB4	-	0.237
**Midkine**	-	0.000
**Myeloperoxidase**	0.315	0.000
**Prolactin**	0.000	-
**Resistin**	0.001	0.001
Substance P		0.220
**Thromboxane B2**	0.009	0.708
TNF receptor 2	0.186	-

Concentration differences of biomarkers in bold are significant in at least one body fluid.

**Table 5:** Overview of biomarkers actively contributing to the AUC of the bio depression score.

	Biomarkers	MDD	Male MDD	Female MDD
Serum				
	α1 anti-trypsin	x	x	x
	BDNF free	x	x	x
	BDNF total	x	x	x
	Calprotectin	x	x	x
	Cortisol	x	x	x
	Prolactin	x	x	x
	Resistin	x	x	x
	Thromboxane	x	x	x
	TNF receptor 2	x	x	x
	**Apolipoprotein A1**	x	x	
	**EGF**	x	x	
	**Myeloperoxidase**	x	x	
	**Endothelin**	x		x
	**Leptin**	x		x
Urine				
	Aldosteron	x	x	x
	α1 anti-trypsin	x	x	x
	Calproctectin	x	x	x
	cGMP	x	x	x
	HVEM	x	x	x
	Isoprostane	x	x	x
	Leptin	x	x	x
	LTB4	x	x	x
	Resistin	x	x	x
	**Cortisol**	x	x	
	**Substance P**	x	x	
	**Thromboxane**	x	x	
	**Myeloperoxidase**	x		x
	**Midkine**			x

Under all conditions BMI is also an active bilmarker; Bold biomarkers are significantly different between men and women.

**Table 6:** Biomarkers tested, the sample origin and manufacturer of the commercially available assay.

	Biomarker	Sample	Manufacturer
1	Aldosterone	Urine	R&D Systems
2	alpha1 anti-trypsin	Serum	Immundiagnostik
3	alpha1 anti-trypsin	Urine	Immundiagnostik
4	Apolipoprotein	Serum	R&D Systems
5	Free BDNF *	Serum	R&D Systems
6	Total BDNF *	Serum	R&D Systems
7	Calprotectin	Urine	Hycult Biotech
8	Calprotectin	Serum	Hycult Biotech
9	cGMP	Urine	R&D Systems
10	Cortisol	Urine	Diagnostics Biochem Canada Inc.
11	Cortisol	Serum	Diagnostics Biochem Canada Inc.
12	EGF	Serum	R&D Systems
13	Endothelin-1	Serum	R&D Systems
14	HVEM	Urine	R&D Systems
15	Isoprostane-2	Urine	Northwest LLC
16	Leptin	Urine	R&D Systems
17	Leptin	Serum	R&D Systems
18	LTB4	Urine	R&D Systems
19	Midkine	Urine	R&D Systems
20	Myeloperoxidase	Serum	R&D Systems
21	Myeloperoxidase	Urine	R&D Systems
22	Prolactin	Serum	Diagnostics Biochem Canada Inc.
23	Resistin	Serum	R&D Systems
24	Resistin	Urine	R&D Systems
25	Substance P	Urine	R&D Systems
26	Thromboxane	Serum	R&D Systems
27	Thromboxane	Urine	R&D Systems
28	TNFα receptor 2	Serum	R&D Systems

* Free BDNF is unbound BDNF in serum only, whereas total BDNF is both free and bound BDNF in serum.

## Supplementary Materials

Supplementary materials can be found at https://www.mdpi.com/1422-0067/21/9/3039/s1. Figure S1 Kaplan-Meier analysis of MDD status irrespective of day of sample drawing; Table S1 Mann whitney U and levene’s test results; Table S2 overview of biological mechanisms of MDD and associated biomarkers; Table S3 Comparison of results with and without gender separation within new cohort.

## Abbreviations

QBP	Quantile-Based Prediction
AUC	Area Under Curve
BMI	Body Mass Index
ROC	Receiver Operator Characteristics
BDS	Bio Depression Score
BDNF	Brain Derived Neurotrophic factor
TNF	Tumor Necrosis Factor
HVEM	Herpesvirus entry mediator
EGF	Endothelin Growth Factor
TMB	Tetramethylbenzidine
