# Supplementary material for: Gender Differences in Developing Biomarker-Based Major Depressive Disorder Diagnostics
Source: Int J Mol Sci. 2020 Apr 25;21(9):3039. doi: 10.3390/ijms21093039 (PMC7246841; doi:10.3390/ijms21093039)

Supplementary Figure S1: Kaplan-Meier analysis of MDD status irrespective of day of sample drawing

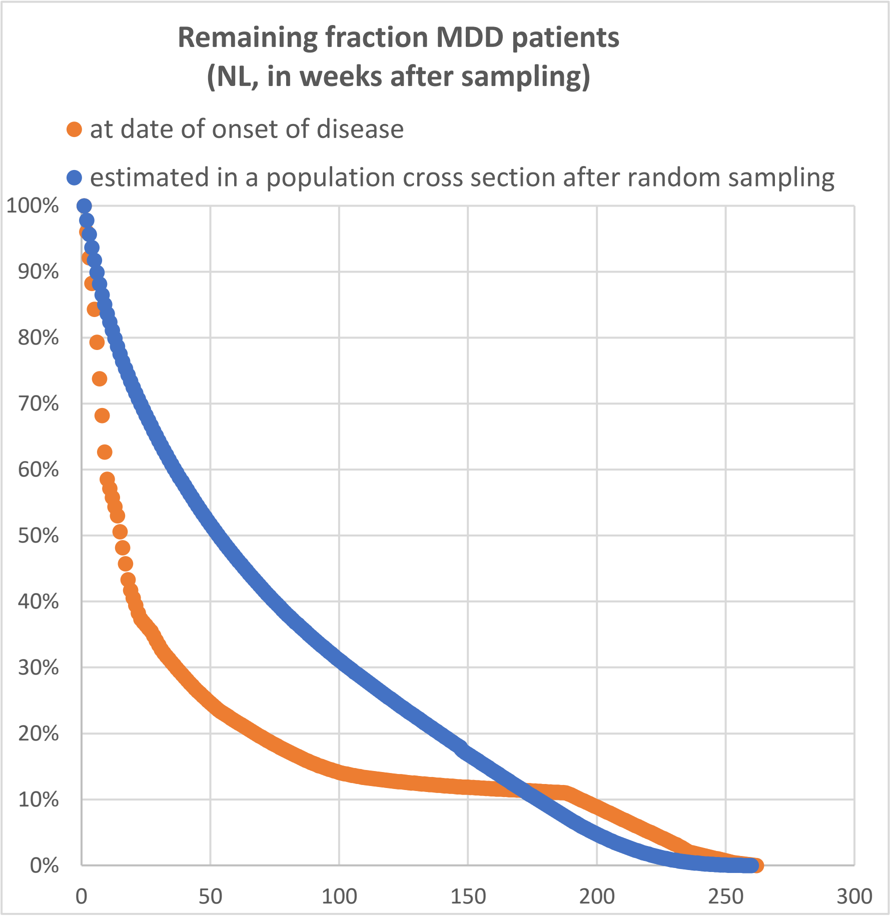

Supplement: Supplementary file 1 [file ijms-21-03039-s001.zip › Figure S1 Kaplan-Meier analysis of MDD status irrespective of day of sample drawing.pdf]
