# Supplementary material for: Gender Differences in Developing Biomarker-Based Major Depressive Disorder Diagnostics
Source: Int J Mol Sci. 2020 Apr 25;21(9):3039. doi: 10.3390/ijms21093039 (PMC7246841; doi:10.3390/ijms21093039)
Supplement: Supplementary file 1 [file ijms-21-03039-s001.zip › Table S1 Mann whitney U and levene test results.pdf]

Supplementary Table S1a. Mann Whitney U and Levene's test results for gender MDD vs controls

		***p* value**			
		**Men (N = 200)**		Woman (N = 200)	
Biomarkers	Matrix	Mann-Whitney U test	Levene's test	Mann-Whitney U test	Levene's test
Aldosterone	Urine	0.051	0.656	0.757	0.673
**α1 anti-trypsin**	Serum	0.248	0.086	0.154	0.345
	Urine	0.191	0.037*	0.414	0.601
**Apolipoprotein A1**	Serum	0.64	0.016*	0.439	0.613
BDNF free	Serum	0.219	0.093	0.234	0.41
**BDNF total**	Serum	0.187	0.011*	0.27	0.946
**Calprotectin**	Serum	0.016 *	0.076*	0.0001 **	0.201
	Urine	0.094 *	0.498	0.092	0.041 *
**cGMP**	Urine	0.024 *	0.014*	0.187	0.498
**Cortisol**	Serum	0.119	0.138	0.126	0.331
	Urine	0.005 **	0.077	0.569	0.939
**EGF**	Serum	0.008 **	0.045*	0.24	0.967
Endothelin	Serum	0.968	0.774	0.218	0.272
**HVEM**	Urine	0.0001 **	0.165	0.0001 **	0.003 **
**Isoprostane**	Urine	0.132	0.692	0.003 **	0.034 *
**Leptin**	Serum	0.485	0.28	0.017 *	0.003 **
	Urine	0.206	0.412	0.209	0.221
LTB4	Urine	0.239	0.154	0.331	0.184
Midkine	Urine	0.734	0.805	0.827	0.678
**Myeloperoxidase**	Serum	0.3	0.589	0.011 *	0.081
	Urine	0.373	0.381	0.164	0.167
**Prolactin**	Serum	0.001 **	0.111	0.95	0.702
**Resistin**	Serum	0.023 *	0.131	0.02 *	0.271
	Urine	0.091	0.155	0.103	0.009 *
Substance P	Urine	0.077	0.252	0.845	0.594
**Thromboxane B2**	Serum	0.014 *	0.147	0.095	0.153
	Urine	0.053	0.422	0.072	0.944
TNF RII	Serum	0.271	0.38	0.083	0.064

* Biomarkers with a $p < 0.05$; ** Biomarkers with a $p < 0.01$

Supplementary Table S1b. Mann-Whitney U test and Levene's test results for case vs control and men vs woman.

		p value			
		Case vs control (N=400)		Men vs woman (N=400)	
**Biomarkers**	**Matrix**	Mann-Whitney U test	Levene's test	Mann-Whitney U test	Levene's test
Aldosterone	Urine	0.356	0.863	3.89E-11 ***	5.02E-07 ***
α1 anti-trypsin	Serum	0.084	0.054	2.006E-07 ***	0.131
	Urine	0.661	0.196	0.013	0.661
Apolipoprotein A1	Serum	0.325	0.137	4.621E-11 ***	0.036 *
BDNF free	Serum	0.095	0.069	0.623	0.567
BDNF total	Serum	0.084	0.104	0.574	0.025*
Calprotectin	Serum	1.41E-05 ***	0.039 *	0.167	0.606
	Urine	0.088	0.055	0 ***	7.525E-08 ***
cGMP	Urine	0.018*	0.179	0 ***	9.604E-07 ***
Cortisol	Serum	0.976	0.047 *	0.045 *	0.017 *
	Urine	0.121	0.481	8.189E-06 ***	0.083
EGF	Serum	0.006 **	0.177	0.283	0.829
Endothelin	Serum	0.396	0.581	0.01 *	0.336
HVEM	Urine	2.81E-07 ***	0.005 **	2.58E-08 ***	0.045 *
Isoprostane	Urine	0.001 **	0.138	0.291	0.018 *
Leptin	Serum	0.084	0.003 **	0 ***	1.588E-14 ***
	Urine	0.121	0.216	0 ***	6.148E-06 ***
LTB4	Urine	0.125	0.071	0.057	0.102
Midkine	Urine	0.911	0.921	3.594E-07 ***	0.026 *
Myeloperoxidase	Serum	0.013 *	0.089	0.47	0.477
	Urine	0.087	0.107	0 ***	0.001 **
Prolactin	Serum	0.024 *	0.725	3.334E-06 ***	0.005 **
Resistin	Serum	0.001 **	0.963	1.186E-06 ***	0.013 *
	Urine	0.028 *	0.005 **	2.402E-07 ***	0.001 **
Substance P	Urine	0.189	0.286	0.004 **	0.212
Thromboxane B2	Serum	0.556	0.934	0.524	0.93
	Urine	0.009 **	0.599	0.806	0.573
TNF RII	Serum	0.045 *	0.075	0.016 *	0.138

* Biomarkers with a $p < 0.05$; ** Biomarkers with a $p < 0.01$; *** Biomarkers with a $p < 0.0001$
