# Supplementary material for: Gender Differences in Developing Biomarker-Based Major Depressive Disorder Diagnostics
Source: Int J Mol Sci. 2020 Apr 25;21(9):3039. doi: 10.3390/ijms21093039 (PMC7246841; doi:10.3390/ijms21093039)
Supplement: Supplementary file 1 [file ijms-21-03039-s001.zip › Table S2 overview of biological mechanisms of MDD and associated biomarkers.pdf]

Table S2 overview of biological mechanisms of MDD and associated biomarkers

Hypothesis	Description	Biomarkers
Immune-inflammation	The immune-inflammation hypothesis of MDD is supported by a fast body of evidence showing that pro-inflammatory factors induce symptoms of depression wereas blockage of cytokines releaves symptoms. Further evidence suggests that inflammation is associated with specific symptom clusters of MDD as well as specific subtypes. Also low grade inflammation within depression is associated with other hypothesis of MDD such as the HPA axis were cortisol is an important factor for regulating the production of cytokines. In turn high levels of cytokines can trigger the HPA axis leading to a disturbed feedback loop with numerous concequences like affecting levels of monoamines such as serotonin	TNF α receptor 2<br>HVEM<br>Calprotectin<br>Substance P<br>Resistin<br>Myeloperoxidase<br>alpha1 anti-trypsin<br>Midkine<br>LTB4<br>Leptin
Neuroplasticity	Numerous studies have shown that neuroplasticity plays a role in MDD. MRI scan showed decreased volumes of the hippocampus which is one of the 2 brain regions were neurogenesis takes place. Also reduced levels of neurotropic factors such as BDNF have been found to be associated with MDD. research suggest that microglia play an important role in altering neuroplasticity within MDD. Microglia are a special class of immune cells scanning the brain for immune related treats and are involved in repairing neuron damage. activated microglia however are associated with neuro-inflammation, reduction of neurotrophic factors and neurodegeneration and become less effective with increasing age leading to an imbalance between neuroprotection and neuroinflammation thereby altering neuroplasticity. Microglia are also sensitive to chronic stress responses	BDNF<br>Midkine<br>TNF α receptor 2<br>EGF<br>Cortisol<br>cGMP

PA axis/ Stress	Stress is next to the monoamine hypothesis one of the oldest hypothesis associated with MDD. Over the years multiple studies have shown that increased levels of cortisol and disturbances of the HPA axis play an important role in certain subtypes of depression such as melancholic depression. Also the HPA axis is connected to multiple mechanisms like immune disturbances, neuroplasticity and monoamine disturbances, playing an important role in the dynamic envirnonment of MDD pathophysiology.	Cortisol<br>Aldosterone<br>Substance P<br>Prolactin
Monoamine	Abnormalities in monoaminergic systems such as sertonin, norepinephrine and dopamine are believed to play a role in MDD. Most early medications for MDD targeted the serotinergic system. However recent evidence suggests that the monoarminergic system only plays part in a complex dynamic system inducing depression	Thromboxane B2<br>Substance P
Endothelial dysfuntion / Oxidative stress	Endothelial dysfunction and oxidative stress are causely related to multiple immune and metabolic processes and most likely play a role in the dynamic process underlying MDD. Induced inflammatory and metabolic processes can lead to a disturbed balance between vasodilating and vasoconstricting factors leading to endothelial dysfunction which in turn can lead to an further increase in inflammatory response and the production of reactive oxygen species which are believed to play a role in neurodegeneration and microglia activation. endothelial dysfunction is believed to play an important role in the association between MDD and myocardial dysfunction.	Endothelin-1<br>Thromboxane B2<br>cGMP<br>LTB4<br>Isoprostane F2<br>Leptin<br>Resistin
Mineral homeostasis	An increasing amount of studies suggest that mineral homeostasis plays an important role in MDD pathophysiology. Minerals play an important part in normal human physiology such as for example iron (Fe) which is essential for succesful binding and transportation of oxygen true the bloodstream. low iron levels however are associated with developing depression. Also disturbances in homeostasis of serveral other minerals such as magnesium (Mg), calcium (Ca), manganese (Mn) and tin (Sn) are associated with MDD. low levels of manganese for example cause depression by inducing auto-immune reactions wereas low levels of tin leads to oxidative stress by disturbing cel protection mechanisms.	Thromboxane B2<br>Aldosterone Substance P
