# Supplementary material for: Gender Differences in Developing Biomarker-Based Major Depressive Disorder Diagnostics
Source: Int J Mol Sci. 2020 Apr 25;21(9):3039. doi: 10.3390/ijms21093039 (PMC7246841; doi:10.3390/ijms21093039)
Supplement: Supplementary file 1 [file ijms-21-03039-s001.zip › Table S3 Comparison of results with and without gender separation within new cohort .pdf]

Table S3 Comparison of results with and without gender separation within new cohort

		QBP		5-fold cross-validation	
		AUC_{real}	spec. at 75% sens	$AUC_{testing}$	spec. at 75% sens
All Ethn	Combined	0.842	82%	0.810	71%
	Female	0.862	90%	0.835	77%
	Male	0.898	93%	0.867	81%
Cauc	Combined	0.845	82%	0.825	77%
	Female	0.905	91%	0.846	81%
	Male	0.891	92%	0.837	74%
